# Land use and land cover change and its impacts on dengue dynamics in China: A systematic review

**DOI:** 10.1371/journal.pntd.0009879

**Published:** 2021-10-20

**Authors:** Panjun Gao, Eva Pilot, Cassandra Rehbock, Marie Gontariuk, Simone Doreleijers, Li Wang, Thomas Krafft, Pim Martens, Qiyong Liu

**Affiliations:** 1 Department of Health, Ethics & Society, CAPHRI Care and Public Health Research Institute, Faculty of Health, Medicine and Life Sciences, Maastricht University, Maastricht, The Netherlands; 2 Key Laboratory of Land Surface Pattern and Simulation, Institute of Geographical Sciences and Natural Resources Research, Chinese Academy of Sciences, Beijing, China; 3 Maastricht Sustainability Institute (MSI), Maastricht University, Maastricht, The Netherlands; 4 State Key Laboratory of Infectious Disease Prevention and Control, Collaborative Innovation Center for Diagnosis and Treatment of Infectious Diseases, National Institute for Communicable Disease Control and Prevention, Chinese Center for Disease Control and Prevention, Beijing, China; Instituto Evandro Chagas, BRAZIL

## Abstract

**Background:**

Dengue is a prioritized public health concern in China. Because of the larger scale, more frequent and wider spatial distribution, the challenge for dengue prevention and control has increased in recent years. While land use and land cover (LULC) change was suggested to be associated with dengue, relevant research has been quite limited. The “Open Door” policy introduced in 1978 led to significant LULC change in China. This systematic review is the first to review the studies on the impacts of LULC change on dengue dynamics in China. This review aims at identifying the research evidence, research gaps and provide insights for future research.

**Methods:**

A systematic literature review was conducted following the PRISMA protocol. The combinations of search terms on LULC, dengue and its vectors were searched in the databases PubMed, Web of Science, and Baidu Scholar. Research conducted on China published from 1978 to December 2019 and written in English or Chinese was selected for further screening. References listed in articles meeting the inclusion criteria were also reviewed and included if again inclusion criteria were met to minimize the probability of missing relevant research.

**Results:**

28 studies published between 1978 and 2017 were included for the full review. Guangdong Province and southern Taiwan were the major regional foci in the literature. The majority of the reviewed studies observed associations between LULC change factors and dengue incidence and distribution. Conflictive evidence was shown in the studies about the impacts of green space and blue space on dengue in China. Transportation infrastructure and urbanization were repeatedly suggested to be positively associated with dengue incidence and spread. The majority of the studies reviewed considered meteorological and sociodemographic factors when they analyzed the effects of LULC change on dengue. Primary and secondary remote sensing (RS) data were the primary source for LULC variables. In 21 of 28 studies, a geographic information system (GIS) was used to process data of environmental variables and dengue cases and to perform spatial analysis of dengue.

**Conclusions:**

The effects of LULC change on the dynamics of dengue in China varied in different periods and regions. The application of RS and GIS enriches the means and dimensions to explore the relations between LULC change and dengue. Further comprehensive regional research is necessary to assess the influence of LULC change on local dengue transmission to provide practical advice for dengue prevention and control.

## Author summary

Dengue is a major public health concern in China. The rapid development of urbanization along with climate change increases the challenge for dengue prevention and control. Previous research has mainly focused on the meteorological variables whereas land use and land cover (LULC) change received comparatively less attention. Our review identified that the regional research hotspots of dengue epidemics in China were Guangdong Province and southern Taiwan. Though inconsistent, most included studies somehow observed associations between at least one of the LULC change factors and dengue. A geographical information system (GIS) was widely used to perform spatial analysis in the selected literature. Its application provided a novel view to describe the relationships between environmental factors and the situation of dengue, which enabled scholars to explore more characteristics of dengue transmission. Meanwhile, the use of remote sensing (RS) enriched the means of environmental monitoring. However, there are research gaps in the area of dengue and LULC change, such as the less consideration of dengue vector study, the lack of interplays between factors, and the lack of considering interventions and policies. Furthermore, because of different research settings, results from these studies were difficult to compare. Thus, further comprehensive and comparable investigations are necessary to better understand the effects of LULC change on dengue in China. This review is the first to expound the studies on the associations between LULC change and dengue dynamics in China. It demonstrates the findings and methodologies and provided insights for future research.

## Introduction

Dengue is currently one of the most important tropical diseases globally, with a 30-fold increase in incidence and a rapid geographical expansion in recent decades [1]. The global economic cost of dengue was estimated as nearly nine billion US dollars [2]. Without an effective vaccine [3], it is urgent to promote adequate surveillance and control of dengue to protect public health and reduce disease burden.

Dengue dynamics, in the perspectives of incidence and distribution of dengue cases and vectors in both temporal and spatial scales, were widely illustrated to be affected by various factors. Land use and land cover (LULC) change has been suggested to be one of the risk factors. Land use usually refers to the human’s utilization of land cover types, while land cover refers to the physical and biological surface of land [4]. The changes in LULC characteristics, such as the types, sizes, and distribution, were suggested to be associated with dengue. Some studies have reported that land cover types were related to the change of dengue incidence [5,6], and others found that LULC change affects the risk of dengue transmission through its impacts on the vector population [7–9].

Significant LULC change has been taking place in China [10], particularly since the economic "Open Door" policy was implemented in 1978. Due to the development of special economic zones following this policy, the accelerated industrialization increased the urbanization ratio in these areas and affected LULC [11]. The last four decades were characterized by rapid and unprecedented urbanization and LULC change all over China. Meanwhile, since the first dengue outbreak in mainland China after China’s founding in 1949 was reported in 1978, China has been through dengue outbreaks of increasingly larger scales and a widening spatial distribution [12,13].

Climate change, risk of imported cases, limited surveillance on dengue vectors, and increasing population migration [13–15], bring more pressure on the prevention and control of dengue in China. Compared to these risk factors, LULC is more alterable and affected by human activity in a relatively short time. Hence, established associations of the cover and utilization of local land with dengue epidemics might be helpful for targeted interventions, especially in regions experiencing LULC change due to urbanization and industrial transformation.

Several investigations have been conducted to study the associations between LULC change and dengue in China, yet an overview is lacking. A comprehensive review of previous work since early years would be helpful to look back and provide insights for future research. To our best knowledge, no previous review studies looked into the effects of LULC change on spatiotemporal dynamics of dengue in China before. Therefore, this review is the first to narrate China’s research progress on this topic systematically. By performing this systematic review, we aimed to address the following research questions: 1) whether effects of LULC change on dengue have been identified in China; 2) whether the recognized effects were consistent within different types of LULC and different studies; 3) what methods have been applied in such studies; 4) whether there were relatively neglected aspects of previous research needing more attention and investigation. This systematic review aimed to identify current research gaps and provide insights on directions and methodologies for future research.

## Methods

This systematic literature search was carried out on 9^th^ December 2019. Therefore, the guidelines of Preferred Reporting Items for Systematic Reviews and Meta-Analyses (PRISMA) [16] published in 2009 were followed. PRISMA helps systematically assess and interpret the selected studies to improve the quality of systematic reviews [16]. This review was conducted to establish a comprehensive understanding of the potential impacts of LULC change on the incidence and distribution of dengue in China, and to identify research gaps and provide insights for future research.

### Definitions of types of land use and land cover

For this study, LULC change was described from four aspects: green space, blue space, transportation infrastructure, and urbanization. The concept of green and blue space and their health effects have been widely discussed in research [17,18]. Hence, we continued with these terminologies and clarified their definitions in this review. Transportation infrastructure was suggested to profoundly influence land use change also with long term impact [19]. Therefore, the land use related to transportation was included in this review. Rapid growth of port cities was considered to be responsible for major dengue epidemics and accelerated urbanization has great impacts on land use change [20–22]. Therefore, the land types related to urbanization were also included in this review. Each specific land type was classified, referring to the Chinese National Standard of Current Land Use Classification (GB/T 21010–2017) and the Law of the People’s Republic of China on Land Administration.

Green space often refers to the natural and semi-natural landscapes of water and vegetation or the urban vegetated areas [23]. Nevertheless, in this review, green space was narrowly defined as vegetated areas regardless of whether they are natural or urban, such as grassland, woodland, and cultivated land. Though waters have been considered a subset of green space, there is a trend separating blue space from the commonly defined green space [17]. For this review, blue space referred to visible surface water areas [17], such as rivers, ponds, lakes, swamps, and wetlands [24]. Instead of movement, transportation infrastructures were involved in this article, including road networks and public transportation service infrastructures, such as subway stations. Urbanization was indicated by LULC-related factors except for road network, even though some studies adopted population characteristics [25–27] as indicators to investigate the relationship between urbanization and dengue. In this review, indicators and land types of urbanization included those related to urban land, construction land, and residential land.

### Search strategy

To cover as many comparative studies as possible, two international databases, PubMed (PMD) and Web of Science (WOS), as well as one Chinese database, Baidu Scholar (BDS), were searched. Articles written in English and Chinese were included. With the introduction of the "Open Door" policy in 1978, a new economic dynamic started to influence LULC in China. Hence, the timeframe for the search conducted was set from 1978 to December 9th, 2019 (date of this literature review) in PMD and BDS. In WOS, articles from 1988 to 2019 were involved because only articles published after 1988 were available in this database. All records gathered from the three databases were included in the screening process.

### Search terms

Based on the elements of an epidemiological study [28], the terms were divided into four groups: Exposure, Subject, Outcome, and Study Area. The search terms of Exposure and Subject were set as limited, while those of Outcome and Study Area were searched as terms from the Medical Subject Headings (MeSH) thesaurus. All of the search terms were listed in S1 Table.

The search terms were combined using Boolean operators and limited to the fields Title/Abstract in PMD and TOPIC in WOS. The Title/Abstract in PMD includes a citation’s title, collection title, abstract, other abstract and keywords. Thus, Title/Abstract in PMD was considered to be broad enough for an effective search. The TOPIC in WOS includes title, abstract, author keywords, and keywords plus. Since both of them cover title, abstract and keywords, PMD and WOS were considered to be comparable. An example of the search strategy in PMD is shown in S2 Table. Due to the linguistic characteristics of Chinese, only the translation and alternative translation of Exposure and Subject terms were searched in the BDS database. Based on the features of this database, each search term of Exposure and Subject was combined, and the search was carried out within all fields.

### Inclusion and exclusion criteria

The inclusion and exclusion criteria applied for the selection process are shown in Table 1. Factors related to LULC change were listed in the Exposure group of S1 Table. By mentioning the "effects" of environmental factors, we specifically meant the correlations and associations from the statistical perspective. Considering the large scale of China and improving the efficiency of this review, studies that could represent the situation of a city at least were included for further screening. To minimize missing any relevant studies, references of all studies included were also reviewed, applying the same inclusion and exclusion criteria. The complete screening process was performed by two authors individually.

**Table 1:** Inclusion and exclusion criteria for article selection.

Inclusion criteria	Exclusion criteria
− Publication:01.01.1978–09.12.2019	− Study on vector habits (such as the types of containers)
− Language: English or Chinese	− Study on vector characteristics (such as behaviours, oviposition)
− Study location: Mainland China, Hong Kong, Macau, or Taiwan	− Study on the types of traps
− Study subjects: dengue or dengue vectors	− Study on vector control or resistance
− Factors studied included at least one factor related to LULC change (S1 Table) − Studied LULC change factors were explicitly stated	− Study on methods of vector surveillance
− The effects of environmental factors on dengue were reported	− Study on imported risk
− The effect direction of LULC change factors was explicitly stated if applicable	− Clinical or preclinical study
− Human and/or vector population study	− Review
− Study can represent the situation of a city or a larger scale	− Study protocol
− Full-text of the article can be accessed	− Transmission risk in blood transfusion
	− Intervention assessment
	− Economic burden
	− Study on ’knowledge, attitude, and practice’
	− Study on perception
	− Dengue virus in other animals not mosquitoes

## Results

### Article selection

**Fig 1:**
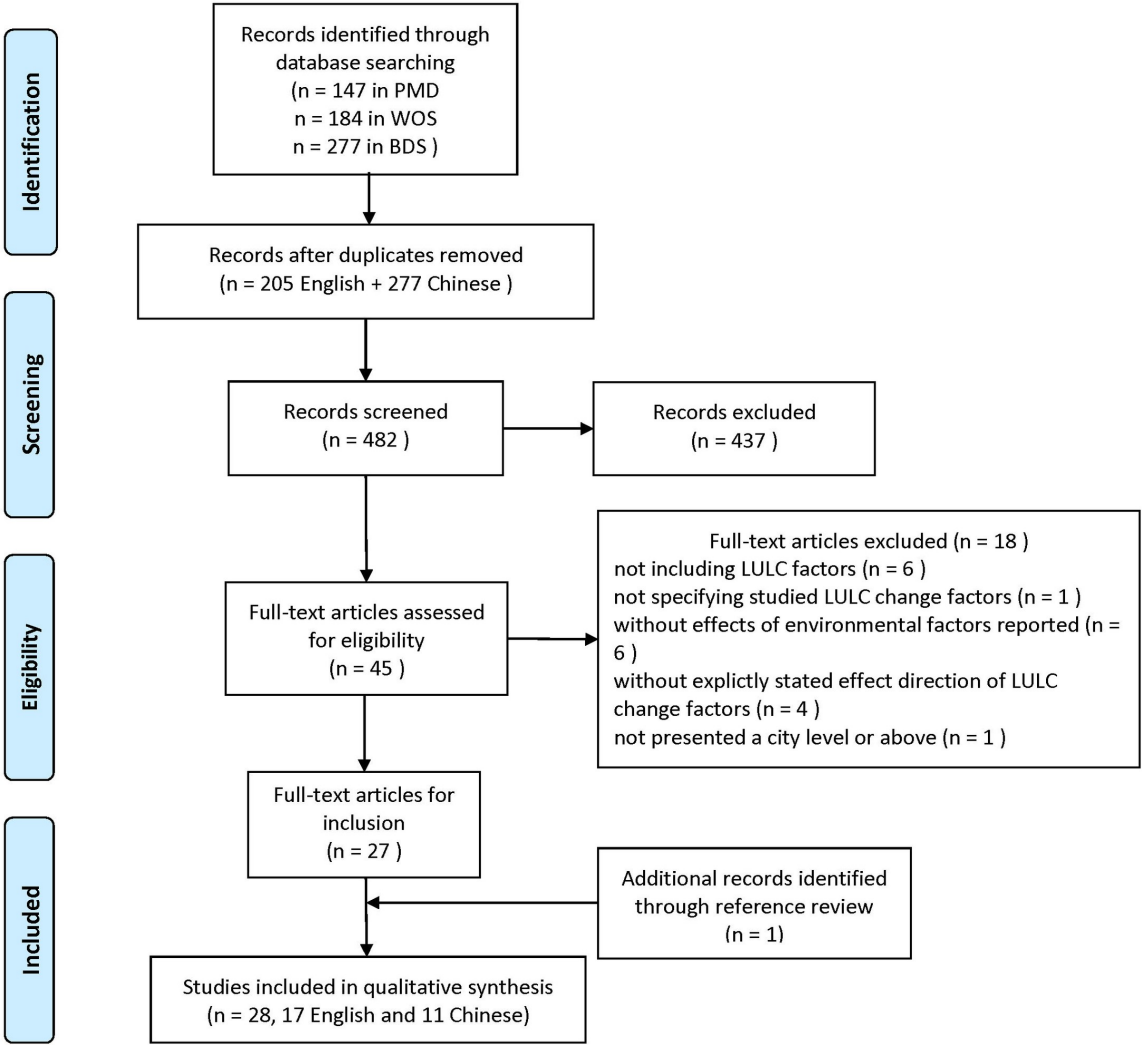
PRISMA flow diagram of the article selection procedure.

The search strategy identified 331 articles in the two international databases and 277 articles in the Chinese database. 205 English and 277 Chinese articles remained after 126 duplicates were removed. Applying the criteria for inclusion and exclusion, 437 articles were excluded after the title and abstract review. 27 articles met the inclusion criteria after the assessment of 45 full-text articles. One additional article was identified after the references of each full-text article were screened. Thus, 28 articles, including 17 in English and 11 in Chinese, were included for further qualitative analysis. The details of the selection process for this systematic review were summarized in the PRISMA flow diagram (Fig 1).

### Characteristics of studies included

Out of 28 selected publications, 23 studies geographically focused on mainland China [29–51] and five on Taiwan [52–56]. The 21 studies on mainland China covered Guangzhou city, Guangdong Province. The study periods ranged from 1978 to 2017. Nine studies focused on the outbreak of dengue in Guangzhou and its surrounding regions in 2014. Five retrospective studies in Taiwan looked at dengue in its southern parts from 1990 to 2015. A complete summary of the included studies and their characteristics is shown in S3 Table, including study period, study area, dengue indicators, source of dengue data, study scale, environmental factors, source of studied factors, data processing, analysis, findings and limitations.

### Roles of land use and land cover change factors

The effects of LULC change factors reported by reviewed literature are shown in Table 2. Three articles used unspecified types of LULC to investigate their effects on dengue. Two of them reported positive impacts on the incidence of dengue [38,50], while the rest found a fluctuant relationship between land cover and dengue incidence [32].

**Table 2:** The effects of LULC change factors on dengue dynamics in selected articles (number of related studies, indicator/land type).

LULC Association		Negative		Positive		Nonlinear		Not significant	
Unspecified LULC		0,	None	2,	Land types including water, vegetation, building [38,50]	1,	Land cover [32]	0,	None
Green space	Vegetation	3,	VFC [29] NDVI [53] EVI [51]	3,	Green cover ratio [54] NDVI [45,49]	5,	NDVI [30,32–34,39]	5,	NDVI [36] NDWI [38,50] Vegetation [35,42]
	Agricultural land	7,	Agricultural land [41,43,44,46,48,52,53]	2,	Agricultural land [30,42]	1,	C3 nitrogen-fixing crops [31] C4 perennial crops [31]	2,	C3 annual crops [31] C3 perennial crops [31] C4 annual crops [31] Agricultural land [37]
	Forest	6,	Forest [41,43,44,46,48,53]	1,	Forest [30]	0,	None	2,	Forested primary land [31] Potentially forested secondary land [31] Forest [37]
	Grassland	2,	Grassland [46,53]	2,	Grassland [30,48]	0,	None	4,	Rangeland [31] mountain and grassland [42] grassland [43,44]
	Park	0,	None	1,	Park [53]	0,	None	2,	Number of parks [56] Public green land [43]
	Orchard	0,	None	1,	Orchard [48]	0,	None	1,	Orchard [42]
	Shrubs	0,	None	0,	None	0,	None	1,	Shrubs [42]
Blue space	Water body	3,	Water [29] Distance to water [47,55]	2,	Water [37,42]	1,	Water land ratio [39]	7,	Water [35,41,44,46,52,53] Small water body density (per km^2^) [51]
	Wetland	2,	Wetland [42,52]	1,	Wetland [30]	0,	None	1,	Wetland [46]
	Rivers	1,	River [48]	0,	None	0,	None	2,	Rivers [43] River density [30]
	Ponds	1,	Ponds [48]	1,	Ponds [43]	0,	None	0,	None
	Reservoir	1,	Reservoir [48]	0,	None	0,	None	0,	None
	Lake	0,	None	0,	None	0,	None	1,	Lake [48]
	Swamp	0,	None	0,	None	0,	None	1,	Swamp [42]
Transportation infrastructure	Road	2,	Distance to roads [40,55]	6,	Road density [29,36,40,41] Roads [43,48]	4,	Road density [30,33,34,39]	3,	Road density [35,39] Roads [47]
	Public transport facilities	0,	None	1,	Bus stop [35]	0,	None	1,	Subway station [35]
Urbanization	Urban land	0,	None	6,	Developed land [30,42] Urban land [46] Urban village [29,34,35] Urban-rural fringe zone [34]	1,	Urban land ratio [39]	4,	Urban land [31,41,42] Land urbanization level [36]
	Construction land	0,	None	7,	Construction land [29,43,44,48,52] Rural residential land [41] Number of houses [56] Business area [52]	0,	None	6,	Construction land [35,46] Rural residential land [41,46] Urban construction land [51] Other construction land except urban and rural [41] Recreation area [52] Number of markets [56] Number of schools [56]
	Unused land	0,	None	1,	Number of vacant grounds [56]	0,	None	5,	Unused land [35,37,42,44,46]

Note: VFC, vegetation fraction; NDVI, normalized difference vegetation index; EVI, enhanced vegetation index; NDWI, normalized difference water index.

#### Green space

26 studies considered green space while they investigated the distribution and incidence of dengue. Overall, the majority of related articles reported negative associations between green space dengue. However, the findings were inconsistent within each type of green space.

The normalized difference vegetation index (NDVI) was mainly used as the indicator of green space [30,32–34,36,39,45,49,53]. There were other studies employing the normalized difference water index (NDWI)[38,50], enhanced vegetation index (EVI) [51], green cover ratio [54], vegetation fraction (VFC) [29], and vegetation [35,38,42,50]. Among studies including vegetation, nonlinear relationships were mostly noticed. Five investigations found different patterns in the nonlinear associations between greenness and the incidence [30,32,33,39] and distribution [34] of dengue. Three studies found negative associations with the risk of dengue and incidence [29,53] and distribution [51]. There were also three other studies identifying positive correlations [45,49,54]. Nevertheless, five investigations did not reveal any relationships between green space and dengue [35,36,38,42,50].

The most investigated type in selected studies was agricultural land [31,37,41–44,46,48,52,53]. Although some research reported no associations [31,37] or positive associations [30,42] between agricultural land, dengue and its vectors, the majority of related studies demonstrated negative associations between agricultural land and the incidence [44,52,53] and distribution [41,43,46,48] of dengue. Moreover, one study reported a nonlinear association between cropland and the distribution of dengue vectors [31].

Nine investigations involved the forest [30,31,37,41,43,44,46,48,53]. There was one study reporting a positive correlation between forest and dengue [30]. However, more studies illustrated the negative correlations between forest and the dynamics of dengue [41,43,44,46,48,53], while two others showed no relationships between them [31,37].

The effects of grassland were explored in seven studies [30,42–44,46,48,53]. Two of those studies involving grassland found a positive relationship with dengue incidence [30] and distribution [46], while another two reported negative associations [46,53]. Most related articles did not identify that grassland was related to dengue [31,42–44].

There were several investigations involving other types of green space, such as public green land [43,53,56], orchard [42,48], and shrubs [42]. One of the three studies involving public green land demonstrated that it might facilitate the spread of dengue [53], while the rest did not observe such effects [43,56]. A positive association between orchard and dengue was found in one of two related studies [48], while no relationship was shown in the other [42]. For shrubs [42], no effects were observed in the included research.

#### Blue space

16 studies considered the potential effects that specific types of blue space may have on dengue transmission. In general, the majority of related studies did not find statistical associations between blue space and dengue. Nevertheless, there were still significant relationships worth noting.

The most studied type of blue space was the water body. Various indicators were applied, including water body areas [29,35,37,41,42,44,46,52,53], water land ratio [39], small water body density [51], and distance to water bodies [47,55]. The effects that blue space may have on dengue were contradictory. Some research uncovered a negative association between water body areas and dengue incidence [29], while others found no relationships [35,41,44,46,52,53] or positive relationships [37,42]. Negative relationships were found between distance to water bodies and dengue incidence and the Breteau Index [47,55], which also suggested the facilitating roles of the water body. Interestingly, one study suggested that surface water area increased the genetic diversity of the dengue virus [37]. It was found that the water land ratio could increase the risk of dengue epidemics within a certain range in the border area of Yunnan and Myanmar [39]. No correlation was shown between small water body density and dengue distribution [51].

The findings on impacts of wetland on dengue were inconsistent. Two related studies reported that wetland was negatively correlated [42,52] to the incidence of dengue. However, two other studies revealed a positive relationship [30] or no relationship [46].

Effects of rivers on dengue dynamics were explored as well. Correlations with dengue were neither identified while using river density as an index [30] nor in one research involving rivers [43]. However, the other research considering rivers reported negative effects on the dengue distribution of rivers [48].

Various effects were found in the two studies looking into the roles of ponds on dengue [43,48]. One of them noticed a negative relationship between ponds and dengue distribution [48], while the other found ponds to be positively associated with the distribution of dengue [43].

The effects of several other types of blue space on dengue were assessed, including reservoir [48], lake [48], and swamp [42]. Negative effects on the dengue distribution of reservoir were reported [48]. Associations with dengue have not been suggested in the studies involving lake [48] or swamp [42].

#### Transportation infrastructure

13 studies investigated the roles of transportation infrastructure on the spread and incidence of dengue. Road and facilities of public transportation were investigated. The facilitating effects of transportation indicators on dengue were relatively consistent despite different study areas and different study periods.

The indicators of road in the selected literature included road density [29,30,33–36,39–41] and distances to roads [40,55]. The incidence and distribution of dengue were positively associated with roads [29,36,40,41,43,48]. At the same time, the increasing distance to roads was negatively associated with dengue [40,55]. Apart from linear relationships, nonlinear associations between road density and dengue dynamics were also noticed. Two studies in the Pearl River Delta region found the first rising and then stable relationships between road density and dengue incidence [30,33]. The association in the border area of Yunnan and Myanmar was found to be rising wave-like [39]. A first decreasing then increasing correlation between road density and dengue distribution was reported in a study carried out in Guangzhou [34]. Another three studies did not find any associations between roads and dengue [35,39,47].

One study looked into the impacts of bus stops and subway stations on dengue transmission [35]. The distribution of dengue was found to be positively associated with the number of bus stops, but not associated with subway stations.

#### Urbanization

18 studies used the LULC-related indicators and land types to represent the urbanization level and evaluated their possible effects on dengue. The majority of related investigations revealed positive or no associations between urbanization and dengue.

The influence of urban land on dengue varied among the reviewed literature. Developed land [30,42] was indicated to increase dengue incidence and urban land increased the risk of extended dengue distribution [46]. One study employing urban land ratio as an indicator found nonlinear associations with dengue incidence in its two considered study areas [39]. Nevertheless, four investigations involving urban land found no direct link to dengue [31,36,41,42]. The effects of the urban village, one of the characteristic land types of Guangzhou, were underlined. The ratio of the urban village was positively associated with the incidence of dengue [29]. Urban village [34,35] and urban-rural fringe zone [34] were positively related to dengue distribution.

The impacts of construction land on dengue dynamics were assessed. In the articles reviewed for this study, the construction land usually referred to the residential area, but there were also studies including commercial area. Higher incidences of dengue [29,44,52] and higher risks of dengue spread [41,43,48,56] were observed to be associated with construction land in seven related studies. The number of houses was suggested to facilitate the spread of dengue [56] as well. However, no effects of construction land on the prevalence or spatial variance of dengue were indicated in six other investigations [35,41,46,51,52,56].

Six studies considered the impacts that unused land might have on dengue. There was only one of them demonstrating the positive effects on dengue spread [56]. The rest did not identify any relationships between unused land and dengue [35,37,42,44,46].

### Roles of other environmental and sociodemographic factors

24 studies included in our review took other environmental and sociodemographic factors into account when investigating the effects of LULC change on dengue. 16 studies involved meteorological factors. Temperature and precipitation were the most widely analyzed variables and were generally found to be positively associated with the incidence and distribution of dengue [29–32,38,39,43,44,48,50,51].

Two of the studies assessed the effects of air pollutants [43,48]. They both studied the dengue situation in Guangzhou in 2014. It was found that fine particulate matter (PM_2.5_) was positively, while carbon monoxide (CO) was negatively associated with dengue distribution. Nevertheless, the effects of sulfur dioxide (SO_2_), nitrogen dioxide (NO_2_), and PM_10_ on the spread of dengue were contradictory.

Population density was used to describe the sociodemographic characteristics of the related study areas. The majority of related studies found population density to be related to a higher risk of dengue and the diffusion of dengue to some degree [29,30,33,38,41,43,44,46,51,54,56]. Gross domestic product (GDP) was the most popular index to indicate the economic condition of the study area. Its impact on dengue dynamics was inconsistent throughout various studies. Some noticed its positive effects [35,36], some negative effects [29,35,36], and some a non-linear relationship between GDP and dengue [33,34], while others found no significant associations [30,38,39,50].

### Methodology on spatial analysis in selected literature

Spatial analysis was widely applied in reviewed studies to identify the clusters of dengue cases and assess LULC change factors’ impacts by spatially locating the dengue cases, calculating dengue incidence at gridded scales, and processing environmental variables. Remote sensing (RS) satellite image was the major source of LULC data in the studies included in this review. LULC data of 13 articles were obtained based on primary and secondary data of Landsat remote sensing satellite imagery; four directly from Landsat products [29,35,37,54], and nine from Resources and Environment Science Data Center of the Chinese Academy of Science (RESDC, CAS) [30,34–36,39–41,44,46]. Eight studies retrieved the data from Moderate Resolution Imaging Spectroradiometer (MODIS) [29,35,37,38,42,50,51,53] and two from Systeme Probatoire d’Observation de la Terre (SPOT) satellite images [43,48].

A geographical information system (GIS) was employed to perform spatial analyses in the majority of the included research except for seven studies [33,37,41,43,48,54,55]. The major platform used was ArcGIS [29–32,35,36,39,40,42,45,47,49,51–53,56]. The majority of included research employed relatively fine spatial scales, such as township or street [29,33,34,54], village [52,53,56], and 1km x 1km– 4km x 4km [30,32,35,36,38,39,41,43,44,46,50].

The effects of LULC types with different RS sources and study scales on dengue are shown in Table 3. This table compares the effects of the same LULC change indicator which was derived from at least two kinds of explicitly stated RS data sources, and those of the same indicator with the same source but different study scales.

**Table 3:** The effects of LULC change indicators with different RS data source or different study scale (number of related studies, effects).

LULC change indicators		Study scale	Landsat	MODIS	SPOT
Green Space	agricultural land	1km x 1km	2, Negative [41,44] 1, Positive [30]	\	1, Negative [48]
		1 km x 1 km - 10km x 10km	1, Negative [46]	\	\
		district	\	1, Positive [42]	1, Negative [48]
	forest	1km x 1km	2, Negative [41,44] 1, Positive [30]	\	1, Negative [43]
		1 km x 1 km - 10km x 10km	1, Negative [46]	\	\
		district	\	\	1, Negative [48]
	grassland	1km x 1km	1, Positive [30] 1, Not significant [44]	\	1, Not significant [43]
		1 km x 1 km - 10km x 10km	1, Negative [46]	\	\
		district	\	\	1, Positive [48]
	orchard	district	\	1, Not significant [42]	1, Positive [48]
Blue space	water	1km x 1km	2, Not significant [41,44]	\	\
		1 km x 1 km - 10km x 10km	1, Not significant [46]	\	\
	wetland	1km x 1km	1, Positive [30]	\	\
		1 km x 1 km - 10km x 10km	1, Not significant [46]	\	\
		district	\	1, Negative [42]	\
	rivers	1 km x 1 km	\	\	1, Not significant [43]
		district	\	\	1, Negative [48]
	ponds	1 km x 1 km	\	\	1, Positive [43]
		district	\	\	1, Negative [48]
Transportation infrastructure	road density	1km x 1km	1, Nonlinear [30]	\	\
		1km x 1km - 6km x 6km	1, Positive [36]	\	\
		township	1, Nonlinear [34]	\	\
		city	1, Positive [40]	\	\
	roads	1 km x 1 km	\	\	1, Positive [43]
		district	\	\	1, Positive [48]
Urbanization	urban land	1km x 1km	1, Not significant [41]	\	\
		1 km x 1 km - 10km x 10km	1, Positive [46]	\	\
		district	\	1, Not significant [42]	\
	developed land	1km x 1km	1, Positive [30]	\	\
		district	\	1, Positive [42]	\
	construction land	1 km x 1 km	1, Positive [44]	\	1, Positive [43]
		1 km x 1 km - 10km x 10km	1, Not significant [46]	\	\
		district	\	\	1, Positive [48]
	rural residential land	1km x 1km	1, Positive [41]	\	\
		1 km x 1 km - 10km x 10km	1, Not significant [46]	\	\
	unused land	1 km x 1 km	1, Not significant [44]	\	\
		1 km x 1 km - 10km x 10km	1, Not significant [46]	\	\

Moran’s I was the most widely used method to detect the clusters of dengue [29,35,36,38,39,43,44,46,48,53,55]. Ecological niche models (ENMs) [29–32], generalized additive models (GAM) [33,34,39], geographically weighted regression (GWR) modelling [35,36,55], ordinary least squares (OLS) regression [38,51,52], and land use regression model [41,46] were the major methods conducted to identify the factors which affected the dynamics of dengue.

### Limitations identified in the research

17 reviewed studies mentioned their research limitations. Among these limitations, some were related to dengue cases, and more were about risk factors and the methodology of their investigations.

26 of reviewed studies used dengue cases to indicate the situation of dengue and most of them referred to the indigenous cases. Authors of three investigations thought that the missing of imported cases [30,32,46] would influence their conclusion to some extent. Besides, the lack of misdiagnosed DF cases [32], the baseline of population immunity [34], and temporal interval during the onset to the diagnosis [36] were also considered to be some of the factors which could probably affect their results.

Aside from the limitations related to dengue cases, several articles also mentioned their inadequacies on risk factors. 12 studies suggested the lack of sufficient vector information in their research [29,31,32,35–37,39,44,52,54–56]. Population characteristics such as education [32], behaviors and habits [44] were missing. Little consideration of policies and interventions on dengue was reported [32,34,36, 39,44,56]. It was also considered better to involve more factors in three investigations [35,41,44]. Moreover, the dynamic condition of the environment was ignored. From the short-term perspective, the variation of temperature in a day was neglected [29]. In the long-term studies, the socio-economic and environmental changes [30,31,34,54] and the changes of the virus population [37] during respective study periods were ignored.

Some selected articles noted the limitations in their research methods. A study on two regional epidemics with different severity resulted in the different fitness of the curve [39]. The design of a relatively short study period limited the effectiveness and generalization of their conclusions [35,44]. Moreover, the lack of comprehensive use of RS images [36], the low spatial resolution of limited RS images [37], spatially uneven weather monitoring stations [54], and low temporal resolution [30] were considered to have impacts on the study conclusions. Two studies mentioned the incapacity of the land use regression model to explain the nonlinearity [41,46] or to consider the temporal distribution of dengue [41]. The authors of three studies thought that necessary further temporal and spatial analyses based on their research settings should have been done [36,39,46].

## Discussion

The associations between LULC change and dengue were mainly investigated in regions covering Guangdong Province, Yunnan Province and southern Taiwan. A few studies focused on the major dengue outbreak in Guangzhou city, Guangzhou Province in 2014. Over 45 thousand people were infected during the dengue outbreak in 2014 [57]. Particularly, Guangdong Province [58] and Yunnan Province [59] are also regions experiencing rapid changes due to the “Open Door” policy. These regions are also the hot spot regions for dengue in China [12,60–62]. Evidence on the effects of LULC change, more specifically, green and blue space on dengue in China is conflicting. In contrast, others, such as the effects of transportation and urbanization on dengue, are consistently suggested to increase the risk of dengue transmission.

The effects of green space were illustrated and also found to be inconsistent in other parts of the world. In an Indonesian study, it was reported that the risk of dengue decreased along with increasing forest cover [63]. In India, increasing distance to the forest was found to be one of the risk factors of dengue occurrence [64]. Moreover, in one study, different results were noticed in various areas of Thailand. Distance to irrigated land was positive with infection risk in one city while it was risky to be both far away and nearby irrigated land in another city [65].

Reported pathways through which green space affect dengue tended to support its facilitating roles, though it is still debatable whether green space affects dengue positively or negatively. Land types of green space may affect dengue by providing shaded areas where the productivity of vector pupal would increase [66]. Likewise, in a study on malaria vectors in China, it was found that deforestation and cultivation, and higher ambient temperature caused by these changes were associated with longer vector survivorship [67].

In terms of water space, it was found that people living near sewers without covers, and discharging wastewater directly to ponds were exposed to a higher risk of dengue in Vietnam [68]. It was reported that the two main dengue vectors, *Aedes aegypti* (*Ae*. *aegypti*) and *Aedes albopictus* (*Ae*. *albopictus*) [3], preferred to choose water containers as habitats [69–71]. Small water storage was suggested to be associated with an increased risk of *Ae*. *Aegypti* [72]. At the same time, dengue incidence was found to be affected by river density in Pakistan [73] and the water level of rivers in the Amazon region [74].

It has been indicated that the characteristics of water in containers, such as surface type and area, depth and cleanness, could affect the occurrence and survival of dengue vectors [71]. However, as far as we know, investigations on the effects of the characteristics of larger water bodies on dengue were missing. Compared to green space, evidence explaining why relatively larger water bodies could influence dengue is absent.

Both green [75] and blue space [76] are affected by the climatic condition. The meteorological factors have been recognized as dominant environmental factors that could affect the dynamics of dengue [77]. This may explain why some of the included studies which also considered climatic variables did not observe distinct effects of green and blue space on dengue.

Some studies reported the effects of transportation on dengue in other places. An investigation in the West Indies suggested that the number of dengue cases increased when the proximity from home addresses of dengue cases to roads became shorter [78]. Globally, models predicting the spread of *Ae*. *aegypti* and *Ae*. *albopictus* showed that highways were one of the high-risk introducing routes and that their niche expansion would spread into climatically suitable urban areas [79].

The transportation networks may increase the risk of dengue spread through garages located along highways [80], trade and travel [81]. Furthermore, minor roads and the extension of the road network were found to affect the dynamics and fragmentation of vegetation [82,83], which probably impact the vectors of dengue by providing habitats.

Urbanization has been recognized as one of the main drivers of epidemic dengue emergence [84]. The proportion of urban area was found to be positively related to dengue incidence in Nepal [85]. Besides, the positive associations of urban villages and urban fringe zones on dengue transmission in China were also found in Thailand [86] and India [87].

There are several pathways by which urbanization increases the risk of dengue. Urbanization could increase the risk of dengue by elevating the human-vector contacting rate [88]. Higher distribution of *Ae*. *aegypti* was also observed in urban areas [89]. It was shown that urban and suburban areas were populated with more vector habitats. These areas were featured by increased adult emergence rate and larval survival rate, short larval development time, and extended life span [71]. A study on other species of mosquitoes also suggested that the urban fringe zone could increase the risk of dengue by providing a large area of suitable breeding sites [90]. LULC changes, such as decreasing water bodies and vegetation, and population shifts that happened in the process of urbanization led to spatiotemporally changed patterns of urban heat islands [91]. The microclimate with increasing surface temperature in the urbanized areas [92] might affect dengue transmission.

Other studies have reported the interactive effects within different land types. Ponds and transportation infrastructure were found to be significant interaction factors that could enhance the effects of other LULC change factors on dengue distribution [93]. Interactive effects were also reported between water facilities, economics and residential area [94]. However, there was little discussion in the reviewed articles about the possible interactions between LULC change factors, other environmental factors, and socioeconomic factors. Moreover, considering the background of global climate change and the dominant roles of climate factors in dengue transmission, the interactive play between climate and LULC change should be noticed for further research.

Only did few of the studies reviewed actually look into dengue vectors. The associations between LULC change and dengue cases have been demonstrated in China. As stated before, there is global evidence supporting that the LULC change may have impacts on dengue vectors. However, the relationship between LULC change and dengue vectors were less investigated in China. Besides, the independent impacts of LULC change on the incidence and distribution of dengue could be explored when considering vector information in the analyses. China has set up a national network of vector surveillance since 2005 [95]. It is suggested to make more use of this system and consider dengue vectors in the future.

The disregard of any prevention and control interventions on vectors and human population constitutes a critical limitation to the conclusions of the reviewed research. In the regions with a high risk of dengue in China, various interventions have been developed and applied [96–101]. The evaluation of the environmental impacts and the ability to control dengue are important tasks for future research.

The development of remote sensing enriches the database of environmental parameters with a larger amount and higher resolution. RS images can provide various environmental information, including meteorological factors and LULC variables at different spatial resolutions, which enriches the dataset and supports the exploration of proper relevant research scales [102]. Making appropriate use of RS helps contribute to an enhanced understanding of dengue dynamics and their associations with LULC change by providing comprehensive data of the environment, including climatic and LULC change factors, while also promoting surveillance in remote areas [103]. Our results underline the importance of selecting the appropriate sensors and scales to identify different effects of LULC change on dengue. Due to higher resolution, SPOT was allowed for higher accuracy identifying vegetation types as compared to Landsat [104]. When calculating NDVI, a high correlation was found between Landsat and SPOT [105], while inconsistent conclusions on the correlations between Landsat and MODIS were reported [105,106].

With the “Open Door” policy and the increasing manifestation of climate change, analyzing dengue in a more comprehensive way that includes the variety of influencing factors has become critical for a successful dengue strategy in China. Thus, spatial analysis enabled researchers to explore the change patterns over time and to further understand the specific regional conditions. Geographic Information Systems that combine and analyze environmental data and other geographically referenced data support the understanding of the environmental factors which affect the prevalence and spatial spread of the disease and its vectors [107–109]. In combination with RS, GIS provides a cost-effective and reliable technique for mapping the areas with a higher risk of transmission and the respective influential variables relevant to vectors and vector-borne diseases [110].

There are a couple of limitations of this review. The categorization of indicators in this review might influence results as presented here. For better description and comparison, we exclusively classified every indicator into one of four categories. However, some indicators could demonstrate more than one type of LULC, such as NDVI and NDWI. The land cover with different values of NDVI and NDWI could be interpreted to be green space or blue space. Moreover, the possible differences in LULC may be neglected. Without further specified descriptions of green space in some of the reviewed articles, it was not possible to subdivide the land types into agricultural land, natural land, artificial land and others. Different subtypes of land may have different pathways to affect dengue dynamics.

## Conclusions

This review is, to the authors’ best knowledge, a first attempt to review research that analyzed the associations between LULC change and dengue dynamics in China. In conclusion, even though most of the studies reviewed reported effects of LULC change, the impact on the prevalence and distribution of dengue in China varied temporally and spatially. Combining the studies does reveal a still ambiguous picture of the actual influence of LULC change on dengue in China. While most studies reviewed underlined the relevance, the actual pathways and critical thresholds remain unclear. The occurrence and spread of dengue result from a complex interplay of various factors such as meteorological variables, air pollutants, socioeconomic status, and demographic characteristics. Therefore, a holistic assessment based on time series analysis is needed at the regional level. Future research is encouraged to take advantage of RS and GIS to support the exploration of the associations between LULC change and dengue. The impacts of LULC on dengue vectors in China require more attention and investigation. And it is suggested to involve the factors of society, environment, and demographics and the policies and interventions when investigating the effects of LULC change on dengue, especially in studies with the long-term setting. By now, comprehensive regional investigations and thorough practical investigations considering more details of LULC are sparse. Therefore, more elaborate future research is required to develop effective prevention and control strategies of dengue taking into consideration urban design and urban planning, especially in the era of climate change.

## Supporting information

S1 Table Search terms. (DOCX)

S2 Table Search strategy example (PubMed). (DOCX)

S3 Table Characteristics of included studies. Note: PRD, Pearl River Delta, including 7 cities (Guangzhou, Shenzhen, Dongguan, Foshan, Zhongshan, Zhuhai, Jiangmen); NIDRIS, National Notifiable Disease Reporting Information System; RESDC, CAS, Resource and Environment Science and Data Center, Chinese Academy of Sciences; NLSC, National Land Surveying and Mapping Center.
(DOCX)
