# Supplementary material for: Land use and land cover change and its impacts on dengue dynamics in China: A systematic review
Source: PLoS Negl Trop Dis. 2021 Oct 20;15(10):e0009879. doi: 10.1371/journal.pntd.0009879 (PMC8559955; doi:10.1371/journal.pntd.0009879)
Supplement: S1 Table — (DOCX) [file pntd.0009879.s001.docx]

| **S1 Table. Search terms** | |
| --- | --- |
| Group | Search terms |
| Exposure | "land type", "land cover", "land use", "landscape", "farm", "farmland", "cultivated", "agriculture", "agricultural", “irrigation”, “dam”, "forest", "deforested", "deforestation", "grass", "grassland", “prairie”, "green", "greenspace", "green space", "greenness", "vegetation", "park", “barren”, "wetland", "swamp", "blue", "bluespace", "blue space", "water", "lake", "river", “canal”, "coastal", “marine”, "road", "street", "highway", "traffic", "transport", "transportation", "urban", "urbanized", "urbanised", "urbanization", "urbanisation", "city" |
| Subject | "dengue", "Aedes" |
| Outcome | distribution, pattern, dynamics, expansion, expand, extension, extend, incidence, occurrence, prevalence, epidemic, endemic, outbreak, emerge, emergence, reemerge, reemergence, appearance, reappearance, density, abundance |
| Study area | China, Chinese, Taiwan, Hong Kong, Macau |
