# Supplementary material for: Land use and land cover change and its impacts on dengue dynamics in China: A systematic review
Source: PLoS Negl Trop Dis. 2021 Oct 20;15(10):e0009879. doi: 10.1371/journal.pntd.0009879 (PMC8559955; doi:10.1371/journal.pntd.0009879)
Supplement: S2 Table — (DOCX) [file pntd.0009879.s002.docx]

| **S2 Table. Search strategy example (PubMed)** | |
| --- | --- |
| Search terms | ("land type" OR "land cover" OR "land use" OR "landscape" OR "farm" OR "farmland" OR "cultivated" OR "agriculture" OR "agricultural" OR “irrigation” OR “dam” OR "forest" OR "deforested" OR "deforestation" OR "grass" OR "grassland" OR “prairie” OR "green" OR "greenspace" OR "green space" OR "greenness" OR "vegetation" OR "park" OR “barren” OR "wetland" OR "swamp" OR "blue" OR "bluespace" OR "blue space" OR "water" OR "lake" OR "river" OR “canal” OR "coastal" OR “marine” OR "road" OR "street" OR "highway" OR "traffic" OR "transport" OR "transportation" OR "urban" OR "urbanized" OR "urbanised" OR "urbanization" OR "urbanisation" OR "city") AND ("dengue" OR (“dengue” AND "Aedes")) AND (distribution OR pattern OR dynamics OR expansion OR expand OR extension OR extend OR incidence OR occurrence OR prevalence OR epidemic OR endemic OR outbreak OR emerge OR emergence OR reemerge OR reemergence OR appearance OR reappearance OR density OR abundance) AND (China OR Chinese OR Taiwan OR Hong Kong OR Macau) |
| Search field | Title/Abstract |
| Time frame | 1978.01.01 to 2019.12.09 |
| Language | English, Chinese |
| Article types | Types excluding “Books and documents”, “Comments”, “Congress”, “Editorial”, “Review”, “Scientific Integrity Review”, and “Systematic Review” |
