# Supplementary material for: Land use and land cover change and its impacts on dengue dynamics in China: A systematic review
Source: PLoS Negl Trop Dis. 2021 Oct 20;15(10):e0009879. doi: 10.1371/journal.pntd.0009879 (PMC8559955; doi:10.1371/journal.pntd.0009879)
Supplement: S3 Table — Note: PRD, Pearl River Delta, including 7 cities (Guangzhou, Shenzhen, Dongguan, Foshan, Zhongshan, Zhuhai, Jiangmen); NIDRIS, National Notifiable Disease Reporting Information System; RESDC, CAS, Resource and Environment Science and Data Center, Chinese Academy of Sciences; NLSC, National Land Surveying and Mapping Center. (DOCX) [file pntd.0009879.s003.docx]

| **S3 Table. Characteristics of included studies** | | | | | | | | | | | | | | |
| --- | --- | --- | --- | --- | --- | --- | --- | --- | --- | --- | --- | --- | --- | --- |
| **Language** | **Author** | **Study period** | **Study area** | **Dengue indicators** | **Dengue source** | **Study scale** | **Environmental factors** | | **Source of studied factors** | **Data processing** | **Analysis** | **Findings** | | **Limitations** |
|  |  |  |  |  |  |  |  |  |  |  |  | **incidence/case number** | **distribution** |  |
| EN | Cao, Z. [1] | Jun 01 2014 - Dec 12 2014 | Guangzhou, Guangdong | DF cases | Guangdong CDC | township/street | LULC factors | VFC | MODIS satellite imagery | VFC was calculated using NDVI in ENVI; gridded levels of LULC factors were obtained using Kriging interpolation in ArcGIS; corrected using Advanced Spaceborne Thermal Emission and Reflection Radiometer Global Digital Evlevation Model (ASTER GDEM) data (http://www.gscloud.cn/) | Moran's I; Geographical detector | negative | associated | inadequate adult mosquito data; not consider the variation of temperature within a day |
|  |  |  |  |  |  |  |  | water body | Engineering Technology and Research Center of Guangdong Geography Census Monitoring and Comprehensive Analysis based on Landsat 8 satellite data |  |  | negative | associated |  |
|  |  |  |  |  |  |  |  | road density | GEOFABRIK (http://www.geofabrik.de/index.html) |  |  | positive | associated |  |
|  |  |  |  |  |  |  |  | the ratio of construction land | Engineering Technology and Research Center of Guangdong Geography Census Monitoring and Comprehensive Analysis based on Landsat 8 satellite data |  |  | positive | associated |  |
|  |  |  |  |  |  |  |  | the ratio of urban village | Landsat8-OLIand Quickbird satellite imagery |  |  | positive | associated |  |
|  |  |  |  |  |  |  | meteorological factors | daily mean temperature | Guangdong Meteorological Service |  |  | positive | associated |  |
|  |  |  |  |  |  |  |  | total precipitation |  |  |  | positive | associated |  |
|  |  |  |  |  |  |  | socioeconomics | population density | Socioeconomic Data and Applications Center |  |  | positive |  |  |
|  |  |  |  |  |  |  |  | GDP | National Bureau of Statistics of China |  |  | negative |  |  |
|  |  |  |  |  |  |  |  |  |  |  |  |  |  |  |
| EN | Li, Q. X. [2] | 2003-2013 | PRD, Guangdong | indigenous DF cases | China CDC | 1km x 1km | LULC factors | monthly NDVI | Level-1 and Atmosphere Archive and Distribution System Web Interface (http://ladsweb.nascom.nasa.gov) | geocoded DF cases were obtained using www.geocoding.cn; gridded levels were calculated using ArcGIS | Maxent model (ENM) | peak at 0.25 |  | without considering the dynamic environmental conditions; without considering imported cases |
|  |  |  |  |  |  |  |  | river density | RESDC, CAS |  |  |  |  |  |
|  |  |  |  |  |  |  |  | road density |  |  |  | rise first then stable at 15km/km2 |  |  |
|  |  |  |  |  |  |  |  | agricultural land |  |  |  | positive |  |  |
|  |  |  |  |  |  |  |  | forest |  |  |  | positive |  |  |
|  |  |  |  |  |  |  |  | grassland |  |  |  | positive |  |  |
|  |  |  |  |  |  |  |  | wetland |  |  |  | positive |  |  |
|  |  |  |  |  |  |  |  | developed land |  |  |  | positive |  |  |
|  |  |  |  |  |  |  | meteorological factors | monthly temperature | National Meteorological Information Center of CMA, China/WorldClim |  |  | peak at 24-25C` |  |  |
|  |  |  |  |  |  |  |  | monthly humidity |  |  |  |  |  |  |
|  |  |  |  |  |  |  |  | monthly precipitation |  |  |  | peak at 720mm and 830mm |  |  |
|  |  |  |  |  |  |  | socioeconomics | population density | RESDC, CAS |  |  | peak at 4000 people/km2 |  |  |
|  |  |  |  |  |  |  |  | GDP |  |  |  |  |  |  |
|  |  |  |  |  |  |  |  |  |  |  |  |  |  |  |
| EN | Liu, B. Y. [3] | 1990-date | mainland China | Occurrence of Ae. aegypti and Ae. albopictus | literature records and GBIF database | 5km x 5km | LULC factors | forested primary land | Land-Use Harmonization (LUH2) database (http://luh.umd.edu/index.shtml) | environmental variables were extracted, resampled and cropped to the geographical area of mainland China in ArcGIS; background points were generated in ArcGIS, multicollinearity test in R | Maxent (ENM); binary models |  |  | limited occurrence records of Ae. Aegypti; without considering possible change of urban extent within the study period |
|  |  |  |  |  |  |  |  | Non-forested primary land |  |  |  |  |  |  |
|  |  |  |  |  |  |  |  | potentially forested secondary land |  |  |  |  |  |  |
|  |  |  |  |  |  |  |  | potentially non-forested secondary land |  |  |  |  |  |  |
|  |  |  |  |  |  |  |  | rangeland |  |  |  |  |  |  |
|  |  |  |  |  |  |  |  | urban land |  |  |  |  |  |  |
|  |  |  |  |  |  |  |  | C3 annual crops |  |  |  |  |  |  |
|  |  |  |  |  |  |  |  | C3 perennial crops |  |  |  |  |  |  |
|  |  |  |  |  |  |  |  | C3 nitrogen-fixing crops |  |  |  |  | rise first then drop |  |
|  |  |  |  |  |  |  |  | C4 annual crops |  |  |  |  |  |  |
|  |  |  |  |  |  |  |  | C4 perennial crops |  |  |  |  | rise first then drop on Ae. Albopictus;rise first then stable on Ae. Aegypti |  |
|  |  |  |  |  |  |  | meteorological factors | mean diurnal range | WorldClim dataset and CGIAR CCAFS |  |  |  | negative |  |
|  |  |  |  |  |  |  |  | temperature seasonality |  |  |  |  | negative |  |
|  |  |  |  |  |  |  |  | maximum temperature of the warmest month |  |  |  |  |  |  |
|  |  |  |  |  |  |  |  | precipitation of the driest month |  |  |  |  | rise then drop |  |
|  |  |  |  |  |  |  |  | precipitation seasonality |  |  |  |  |  |  |
|  |  |  |  |  |  |  |  | precipitation of the warmest month |  |  |  |  | positive |  |
|  |  |  |  |  |  |  |  |  |  |  |  |  |  |  |
| EN | Liu, K. K. [4] | 2005-2017 | Guangdong | indigenous DF cases | NIDRIS, China CDC | 1km x 1km | LULC factors | NDVI | geospatial data cloud: www.gscloud.cn | DF cases were aggregated and spatially located in ArcGIS | spatio-temporal clusters of DF incidence analyzed in SaTScan; overall and localized spatial clustering patterns of DF were analyzed; ENM analysis | positive then negative |  | missing unreported DF cases; misdiagnosed DF cases; lack of vector density |
|  |  |  |  |  |  |  |  | Land cover | European space agency |  |  | fluctuant |  |  |
|  |  |  |  |  |  |  | meteorological factors | annual average relative humidity | China meteological data sharing service system |  |  | positive then negative |  |  |
|  |  |  |  |  |  |  |  | average precipitation |  |  |  | positive |  |  |
|  |  |  |  |  |  |  |  | average temperature |  |  |  | positive then stable |  |  |
|  |  |  |  |  |  |  |  | average maximum temperature |  |  |  | positive then stable |  |  |
|  |  |  |  |  |  |  |  | average minimum temperature |  |  |  | positive then stable |  |  |
|  |  |  |  |  |  |  | geographic factors | altitude data | Institute of Geographic Sciences and Natural Resources Research, CAS |  |  | negative |  |  |
|  |  |  |  |  |  |  | socioeconomics | population size | National Bureau of Statistics of China |  |  |  |  |  |
|  |  |  |  |  |  |  |  |  |  |  |  |  |  |  |
| EN | Qi, X. P. [5] | 2013 | PRD, Guangdong | DF cases | NIDRIS, China CDC | township/street | LULC factors | NDVI | Institute of Geographic Sciences and Natural Resources Research, CAS |  | GAM | rise till 0.65 then drop |  | ecological bias; lack of vector density, policy and interventions, education |
|  |  |  |  |  |  |  |  | road density | not mentioned |  |  | rise then stable at 0.4 to 0.7 then rise |  |  |
|  |  |  |  |  |  |  | socioeconomics | urban & rural | National Bureau of Statistics of China |  |  | positive |  |  |
|  |  |  |  |  |  |  |  | population density |  |  |  | drop till 30k-40k then rise |  |  |
|  |  |  |  |  |  |  |  | GDP per capita |  |  |  | fluctuate till 400k CNY, decline after 600k CNY |  |  |
|  |  |  |  |  |  |  | geographic factors | boundary: street/town at the prefectural boundary or not |  |  |  | positive |  |  |
|  |  |  |  |  |  |  |  |  |  |  |  |  |  |  |
| EN | Qu, Y. B. [6] | 2014 | Guangzhou, Guangdong | DF cases | China CDC | township | LULC factors | NDVI | high-resolution, remote-sensing satellite images |  | space-time scan statistical analysis in SaTScan; GAM |  | drop rapidly after 0.25 | lack of historical time series of socio-economic and environmental variables; lack of population immunity, interventions |
|  |  |  |  |  |  |  |  | road density | RESDC, CAS |  |  |  | drop till 50 then rise |  |
|  |  |  |  |  |  |  |  | urban village |  |  |  |  | positive |  |
|  |  |  |  |  |  |  |  | urban-rural fringe zone |  |  |  |  | positive |  |
|  |  |  |  |  |  |  | socioeconomics | population density |  |  |  |  |  |  |
|  |  |  |  |  |  |  |  | GDP per capita | National Bureau of Statistics of China |  |  |  | fluctuant and drop after 800k CNY |  |
|  |  |  |  |  |  |  |  |  |  |  |  |  |  |  |
| EN | Ren, H. Y. [7] | 2012-2014, 2017 | 4 central districts of Guangzhou, Guangdong | DF cases | NIDRIS | 1km x 1km | LULC factors | bus stop | RESDC, CAS | DF cases were geocoded in www.gpsspg.com/xGeocoding/; spatial point layer was produced in ArcGIS | Moran's I; GWR modeling |  | positive | lack of enough factors to explain the spatial variation; lack of enough reliable monitoring data on vector population or density; relatively short time series of DF cases and corresponding remote sensing images |
|  |  |  |  |  |  |  |  | subway station |  |  |  |  |  |  |
|  |  |  |  |  |  |  |  | normal construction land (NCL) | MODIS data, Landsat-5 thematic mapper, Landsat-8 thermal infrared sensor |  |  |  |  |  |
|  |  |  |  |  |  |  |  | urban village(UV) |  |  |  |  | positive |  |
|  |  |  |  |  |  |  |  | water |  |  |  |  |  |  |
|  |  |  |  |  |  |  |  | vegetation |  |  |  |  |  |  |
|  |  |  |  |  |  |  |  | unused land |  |  |  |  |  |  |
|  |  |  |  |  |  |  |  | road density | RESDC, CAS |  |  |  |  |  |
|  |  |  |  |  |  |  | socioeconomics | population density |  |  |  |  |  |  |
|  |  |  |  |  |  |  |  | GDP |  |  |  |  | positive in some regions and negative in others |  |
|  |  |  |  |  |  |  |  |  |  |  |  |  |  |  |
| EN | Ren, H. Y. [8] | 2014 | Guangzhou and Foshan, Guangdong | indigenous DF cases | NIDRIS | 1km x 1km - 6km x 6km | LULC factors | NDVI | ladsweb.nascom.nasa.gov/data | DF cases were spatially located in www.gpsspg.com/xGeocoding/; DF incidence rates were calculated and smoothed at gridded scales in Geoda; spatial grids were created in ArcGIS | Moran's I (best performance at 2km x 2km scale); GWR modeling in ArcGIS |  |  | suitability of 2km x 2km needs to be further assessed; lack of exhausted use of remote sensing images; lack of vector density, interventions, temporal interval during the onset to the diagnosis of each infected patient |
|  |  |  |  |  |  |  |  | road density | RESDC, CAS |  |  |  | positive |  |
|  |  |  |  |  |  |  |  | land urbanization level (LUL) |  |  |  |  |  |  |
|  |  |  |  |  |  |  | meteorological factors | mean temperature (MT) | China meteological data service system |  |  |  |  |  |
|  |  |  |  |  |  |  |  | mean precipitation (MP) |  |  |  |  |  |  |
|  |  |  |  |  |  |  |  | mean relative humidity (MRH) |  |  |  |  |  |  |
|  |  |  |  |  |  |  | socioeconomics | population size | RESDC, CAS |  |  |  | positive |  |
|  |  |  |  |  |  |  |  | GDP |  |  |  |  | negative in some central zones and positive in other zones |  |
|  |  |  |  |  |  |  |  |  |  |  |  |  |  |  |
| EN | Tian, H. Y. [9] | Jan 1978 - Oct 2014 | Guangzhou, Guangdong | notified dengue cases (1978-2014) | notifiable infectious disease report system at Guangzhou CDC | city | LULC factors | cropland | Landsat images and MODIS images,Earth Explorer from Level 1 Product Generation System, Google Earth | possible influence of seasonality of water area was reduced using Enhanced Spatial and Temporal Adaptive Reflectance Fusion Model (ESTAREM); land cover types were classified using iterative self-organizing data analysis techniques algorithm (ISODATA) | phylogenetic analysis; environment-based dengue transmission model |  |  | lack of early data on vector surveillance; complex demographic trends may be ignored when estimating virus population; lack of house-based water surfaces due to lower spatial resolution of limited remote sensing images |
|  |  |  |  |  |  |  |  | forest |  |  |  |  |  |  |
|  |  |  |  | entomological data (Jan 2006-Sep 2014) | notifiable infectious disease report system at Guangzhou CDC |  |  | bare land |  |  |  |  |  |  |
|  |  |  |  |  |  |  |  | water |  |  |  | consistency with virus population shift |  |  |
|  |  |  |  | complete DENV envelope (E) gene (1978-2014) | GenBank |  |  |  |  |  |  | positive with number of DF cases |  |  |
|  |  |  |  |  |  |  | meteorological factors | monthly average values of climatic variables (1978-2014) | local meteorological stations |  |  | annual precipitation was not associated with DF outbreaks |  |  |
|  |  |  |  |  |  |  |  |  |  |  |  |  |  |  |
| EN | Yue, Y. J. [10] | Jan 2014-Dec 2014 | 5 districts of Guangzhou, Guangdong | DF cases | NIDRIS | 1km x 1km | LULC factors | NDWI | image interpretation of GF-1 multi-band remote sensing images from China Center for Resources Satellite Data and Application | DF cases at gridded scales were calculated in ArcMap | spatial patterns were obtained by spatial analysis methods of "point density", "average nearest neighbor"; global Moran's I; host spot analysis (Getis-Ord Gi*); OLS |  |  |  |
|  |  |  |  |  |  |  |  | land types (LT): water, vegetation, buildings |  |  |  | positive |  |  |
|  |  |  |  |  |  |  | meteorological factors | land surface temperature of daytime (LSTD) | MODIS MOD11A2 daily reprocessing products from NASA MODIS LAADS DAAC |  |  |  |  |  |
|  |  |  |  |  |  |  |  | land surface temperature of nighttime (LSTN) |  |  |  | positive |  |  |
|  |  |  |  |  |  |  | socioeconomics | population density (PD) | Institute of Geographic Sciences and Natural Resources Research, CAS |  |  | positive |  |  |
|  |  |  |  |  |  |  |  | GDP |  |  |  |  |  |  |
|  |  |  |  |  |  |  |  |  |  |  |  |  |  |  |
| EN | Zheng, L. [11] | 2010-2014 | PRD, Guangdong | indigenous DF cases | NIDRIS | 1km x 1km-14km x 14km | LULC factors | NDVI | ladsweb.nascom.nasa.gov/data | DF cases were geocoded in www.gpsspg.com/xGeocoding/ | Moran's I in ArcGIS; GAM | "M" shape, risk > 0 between 0.17 and 0.76 |  | the severity of two regional epidemics differed; without considering vector and interventions; spatial correlation was not considered properly; without carefully considering the lagging effect and temporal effect |
|  |  |  |  |  |  |  |  | road density | OpenStreetMap |  |  |  |  |  |
|  |  |  |  |  |  |  |  | land use data | RESDC, CAS |  |  | ULR(urban land ratio): wave rise |  |  |
|  |  |  |  |  |  |  | meteorological factors | mean temperature (Temp) | China Meteorological Data Service Center |  |  | risk > 0 below 18.5 ℃ or above 23.7 ℃ |  |  |
|  |  |  |  |  |  |  |  | mean relative humidity (Hum) |  |  |  | "M" shape, risk > 0 between 79.2% and 82.5% |  |  |
|  |  |  |  |  |  |  |  | mean precipitation (Pre) |  |  |  |  |  |  |
|  |  |  |  |  |  |  | socioeconomics | population size | RESDC, CAS |  |  | rise |  |  |
|  |  |  |  |  |  |  |  | GDP |  |  |  |  |  |  |
|  |  |  | BYM, Yunnan |  |  |  | LULC factors | NDVI | ladsweb.nascom.nasa.gov/data |  |  | "M" shape, risk > 0 between 0.39 and 0.61 |  |  |
|  |  |  |  |  |  |  |  | road density | OpenStreetMap |  |  | wave rise, positive |  |  |
|  |  |  |  |  |  |  |  | land use data | RESDC, CAS |  |  | ULR: risk peak at 0.07 and 0.3; WLR(water land ratio): risk > 0 between 0.15 and 0.20 |  |  |
|  |  |  |  |  |  |  | meteorological factors | mean temperature (Temp) | China Meteorological Data Service Center |  |  | rise, risk > 0 after 17.8 ℃ |  |  |
|  |  |  |  |  |  |  |  | mean relative humidity (Hum) |  |  |  |  |  |  |
|  |  |  |  |  |  |  |  | mean precipitation (Pre) |  |  |  | rise, risk >0 at around 180mm |  |  |
|  |  |  |  |  |  |  | socioeconomics | population size | RESDC, CAS |  |  |  |  |  |
|  |  |  |  |  |  |  |  | GDP |  |  |  |  |  |  |
|  |  |  |  |  |  |  |  |  |  |  |  |  |  |  |
| EN | Li, Q. X. [12] | Jan 26 2014 - Dec 21 2014 | Guangzhou and Foshan, Guangdong | DF cases | NIDRIS | city | LULC factors | distance to the closest road | road network data from RESDC, CAS | DF cases were displayed and geocoded in ArcGIS | zonal statistics; distance analysis | negative |  |  |
|  |  |  |  |  |  |  |  | road density |  |  |  | positive |  |  |
|  |  |  |  |  |  |  |  |  |  |  |  |  |  |  |
| EN | Chuang, T. W. [13] | 2015 | metropolitan area of Tainan,Taiwan | indigenous DF cases | Taiwan CDC | distribution at basic statistical area (BSA) | LULC factors | agriculture | NLSC | the percentages of LULC types were calculated; map layouts were made in ArcGIS | space-time permutation model was performed in SaTScan; OLS regression; spatial regression models were performed in Geoda | negative |  | lack of regular vector surveillance data |
|  |  |  |  |  |  |  |  | wetland |  |  |  | negative |  |  |
|  |  |  |  |  |  |  |  | water |  |  |  |  |  |  |
|  |  |  |  |  |  | LULC analysis at about village level |  | residential area |  |  |  | positive |  |  |
|  |  |  |  |  |  |  |  | recreation area |  |  |  |  |  |  |
|  |  |  |  |  |  |  |  | business area |  |  |  | positive |  |  |
|  |  |  |  |  |  |  |  |  |  |  |  |  |  |  |
| EN | Huang, C. C. [14] | 2014-2015 | rural and urban areas in Tainan, Kaohsiung, Pingtung, Taiwan | DF cases | Taiwan CDC | village | LULC factors | NDVI | MODIS |  | Moran's I in ArcGIS; high/low clustering in ArcGIS; hot spot analysis in ArcGIS; Spearman's rho; generalized linear mixed models (GLMMs); stratified analysis | negative |  |  |
|  |  |  |  |  |  |  |  | farm | NLSC |  |  | negative |  |  |
|  |  |  |  |  |  |  |  | forest |  |  |  | negative |  |  |
|  |  |  |  |  |  |  |  | park |  |  |  | positive |  |  |
|  |  |  |  |  |  |  |  | grassland |  |  |  | negative |  |  |
|  |  |  |  |  |  |  |  | water body area |  |  |  |  |  |  |
|  |  |  |  |  |  |  | socioeconomics | 12 socioeconomic factors | Fiscal Information Agency of the Ministry of Finance in Taiwan |  |  |  |  |  |
|  |  |  |  |  |  |  |  |  |  |  |  |  |  |  |
| EN | Chen, T. H. [15] | 1998-2015 | southern Taiwan: tropical zone between 21°53'50"-23°18'20"N latitude, Taiwan | DF cases | National Infectious Diseases Notification Surveillance System, Taiwan CDC | township level | LULC factors | green cover ratio | satellite images by Landsat TM, ETM+, and TIRS | meteorological data were processed using inverse distance weighting (IDW) and k-nearest neighbors algorithm | negative binomial multilevel approach | positive |  | weather monitoring stations used are spatially uneven; lack of vector abundance data; assuming that socio-ecological factors were stable |
|  |  |  |  |  |  |  | meteorological factors | accumulated most suitable temperature (MST) | Central Weather Bureau of Taiwan |  |  | positive |  |  |
|  |  |  |  |  |  |  |  | dryness in the pre-epidemic period |  |  |  | negative |  |  |
|  |  |  |  |  |  |  |  | cumulative precipitation in the pre-epidemic period |  |  |  | negative |  |  |
|  |  |  |  |  |  |  |  | low precipitation frequency in the epidemic period |  |  |  | negative |  |  |
|  |  |  |  |  |  |  |  | medium precipitation frequency in the epidemic period |  |  |  | positive |  |  |
|  |  |  |  |  |  |  |  | heavy precipitation frequency in the epidemic period |  |  |  |  |  |  |
|  |  |  |  |  |  |  | socioeconomics | population density | Department of Statistics at the Ministry of Interior Affairs |  |  | positive |  |  |
|  |  |  |  |  |  |  |  | old houses | Taiwan Population and Housing Census of 2010 |  |  | negative |  |  |
|  |  |  |  |  |  |  |  |  |  |  |  |  |  |  |
| EN | Hsueh, Y. H. [16] | 2003-2008 | Kaohsiung, Taiwan | DF cases | Health Bureau of Kaoshiung City | Li | LULC factors | distance to major transportation arteries | not mentioned | DF cases were spatially located | Moran's I; G-statistics; GWR | 2004, 2006, 2007: negative |  | lack of vector information |
|  |  |  |  |  |  |  |  | distance to water bodies |  |  |  | negative |  |  |
|  |  |  |  |  |  |  | socioeconomics | population counts | Census 2000 in Taiwan |  |  |  |  |  |
|  |  |  |  |  |  |  |  | population density |  |  |  |  |  |  |
|  |  |  |  |  |  |  |  |  |  |  |  |  |  |  |
| EN | Wen, T. H. [17] | Jun 2007-Jan 2008 | Tainan City, Taiwan | DF cases | Taiwan CDC | village | LULC factors | the number of parks | not mentioned | DF cases were mapped in ArcGIS | exploratory space-time analysis of the spreading dynamics; network analysis of pairs of space-time distances; time-to-event analysis of geographical diffusions |  |  | lack of vector information; assuming that DF cases were infected at workplaces or residences; without considering interventions |
|  |  |  |  |  |  |  |  | the number of schools |  |  |  |  |  |  |
|  |  |  |  |  |  |  |  | the number of markets |  |  |  |  |  |  |
|  |  |  |  |  |  |  |  | the number of vacant grounds |  |  |  |  | positive |  |
|  |  |  |  |  |  |  |  | the number of houses |  |  |  |  | positive |  |
|  |  |  |  |  |  |  | socioeconomics | population density |  |  |  |  | positive |  |
|  |  |  |  |  |  |  |  |  |  |  |  |  |  |  |
| CN | Zheng, L. [18] | Sep 2009 | Guangzhou, Guangdong | DF cases | NIDRIS | 1km x 1km | LULC factors | road density | not mentioned |  | land use regression model |  | positive | lack of enough factors such as weather, height of buildings; land use regression model could not explain the nonlinear relationship between factors and DF; land use regression model could not fully consider the temporal distribution |
|  |  |  |  |  |  |  |  | agricultural land | RESDC, CAS |  |  |  | negative |  |
|  |  |  |  |  |  |  |  | forest |  |  |  |  | negative |  |
|  |  |  |  |  |  |  |  | water |  |  |  |  |  |  |
|  |  |  |  |  |  |  |  | urban land |  |  |  |  |  |  |
|  |  |  |  |  |  |  |  | rural residential land |  |  |  |  | positive |  |
|  |  |  |  |  |  |  |  | other construction land |  |  |  |  |  |  |
|  |  |  |  |  |  |  | socioeconomics | population density |  |  |  |  | positive |  |
|  |  |  |  |  |  |  | entomological factors | BI | not mentioned |  |  |  |  |  |
|  |  |  |  |  |  |  |  |  |  |  |  |  |  |  |
| CN | Li, S. [19] | 2012 | Guangzhou, Guangdong | DF cases | Guangdong CDC | district | LULC factors | agricultural land | MODIS-VI (205M) | the centroid of each district was calculated in ArcGIS | cluster analysis in SaTScan; generalized linear model; logistic regression model | positive |  |  |
|  |  |  |  |  |  |  |  | mountain and grassland |  |  |  |  |  |  |
|  |  |  |  |  |  |  |  | shrubs |  |  |  |  |  |  |
|  |  |  |  |  |  |  |  | orchard |  |  |  |  |  |  |
|  |  |  |  |  |  |  |  | vegetable land |  |  |  |  |  |  |
|  |  |  |  |  |  |  |  | swamp |  |  |  |  |  |  |
|  |  |  |  |  |  |  |  | wetland |  |  |  | negative |  |  |
|  |  |  |  |  |  |  |  | open water area |  |  |  | positive |  |  |
|  |  |  |  |  |  |  |  | urban land |  |  |  |  |  |  |
|  |  |  |  |  |  |  |  | developed land |  |  |  | positive |  |  |
|  |  |  |  |  |  |  |  | unmarked land |  |  |  |  |  |  |
|  |  |  |  |  |  |  |  |  |  |  |  |  |  |  |
| CN | Chen, Y. B. [20] | Jan 2014 -Nov 2014 | 7 districts in Guangzhou, Guangdong | DF cases | Guangdong CDC | 1 km x 1 km | LULC factors | agricultural land | vector data of base map and 2.5m SPOT |  | Global Moran's I; Random Forest |  | negative |  |
|  |  |  |  |  |  |  |  | forest |  |  |  |  | negative |  |
|  |  |  |  |  |  |  |  | grassland |  |  |  |  |  |  |
|  |  |  |  |  |  |  |  | public green land |  |  |  |  |  |  |
|  |  |  |  |  |  |  |  | rivers |  |  |  |  |  |  |
|  |  |  |  |  |  |  |  | ponds |  |  |  |  | positive |  |
|  |  |  |  |  |  |  |  | roads |  |  |  |  | positive |  |
|  |  |  |  |  |  |  |  | residential land |  |  |  |  | positive |  |
|  |  |  |  |  |  |  | meteorological factors | precipitation | Water Resources Department of Guangdong |  |  |  | positive |  |
|  |  |  |  |  |  |  |  | temperature |  |  |  |  | positive |  |
|  |  |  |  |  |  |  | air pollutants | SO2 | Agency of Environmental Protection of Guangzhou |  |  |  |  |  |
|  |  |  |  |  |  |  |  | NO2 |  |  |  |  | positive |  |
|  |  |  |  |  |  |  |  | PM10 |  |  |  |  | positive |  |
|  |  |  |  |  |  |  |  | PM2.5 |  |  |  |  | positive |  |
|  |  |  |  |  |  |  |  | CO |  |  |  |  | negative |  |
|  |  |  |  |  |  |  |  | O3 |  |  |  |  | negative |  |
|  |  |  |  |  |  |  | socioeconomics | population density | Yearbook of Guangzhou 2014 |  |  |  | positive |  |
|  |  |  |  |  |  |  |  |  |  |  |  |  |  |  |
| CN | Ren, H. Y. [21] | 2014 | Guangzhou and Foshan, Guangdong | DF cases | NIDRIS | 1 km x 1 km | LULC factors | agricultural land | RESDC, CAS | DF cases were spatially located and calculated at gridded scale in www.gpsspg.com/xGecoding | global Moran's I in Geoda; back propagation neural network model | negative |  | lack of vector density, road network, interventions, behaviors and habits; the generalization of results was limited by the short study period; the temporal resolution was relatively low |
|  |  |  |  |  |  |  |  | forest |  |  |  | negative |  |  |
|  |  |  |  |  |  |  |  | grassland |  |  |  |  |  |  |
|  |  |  |  |  |  |  |  | water |  |  |  |  |  |  |
|  |  |  |  |  |  |  |  | construction land (urban and rural residential land) |  |  |  | positive |  |  |
|  |  |  |  |  |  |  |  | unused land |  |  |  |  |  |  |
|  |  |  |  |  |  |  | meteorological factors | temperature |  |  |  | positive |  |  |
|  |  |  |  |  |  |  |  | humidity |  |  |  |  |  |  |
|  |  |  |  |  |  |  |  | precipitation |  |  |  | positive |  |  |
|  |  |  |  |  |  |  | socioeconomics | population density |  |  |  | positive |  |  |
|  |  |  |  |  |  |  |  |  |  |  |  |  |  |  |
| CN | Yi, B. T. [22] | 1995 | Guangdong | DF cases | Guangdong CDC | province | LULC factors | NDVI | NOAA-14, obtained from website of LP-DAAC (Land Processes Distributed Archieve Center) | layouts of BI were made in ArcGIS | Co-Kriging model in ArcGIS |  | positive |  |
|  |  |  |  | BI |  |  |  |  |  |  |  |  | positive |  |
|  |  |  |  |  |  |  |  |  |  |  |  |  |  |  |
| CN | Zheng, L. [23] | 2010-2014 | PRD, Guangdong | indigenous DF cases | NIDRIS | 1 km x 1 km - 10km x 10km | LULC factors | agricultural land | RESDC, CAS | DF cases were spatially located in www.geocoding.com | SLEUTH model; land use regression model; Moran's I |  | negative | predictive model needed to be further optimized for short-term prediction; land use regression model could not explain the nonlinear relationship between factors and DF; without considering imported DF cases |
|  |  |  |  |  |  |  |  | forest |  |  |  |  | negative |  |
|  |  |  |  |  |  |  |  | grassland |  |  |  |  | negative |  |
|  |  |  |  |  |  |  |  | wetland |  |  |  |  |  |  |
|  |  |  |  |  |  |  |  | water |  |  |  |  |  |  |
|  |  |  |  |  |  |  |  | urban land |  |  |  |  | positive |  |
|  |  |  |  |  |  |  |  | rural residential land |  |  |  |  |  |  |
|  |  |  |  |  |  |  |  | construction land |  |  |  |  |  |  |
|  |  |  |  |  |  |  |  | unused land |  |  |  |  |  |  |
|  |  |  |  |  |  |  | socioeconomics | population density |  |  |  |  | positive |  |
|  |  |  |  |  |  |  |  |  |  |  |  |  |  |  |
| CN | Yi,B. T. [24] | 1997, 2000 | Chaozhou, Guangdong | BI | Chaozhou CDC | district | LULC factors | roads | 1: 50000 digital maps | surveillance data were spatially located in ArcGIS | spatial cluster analysis in SaTScan; Kriging interpolation; distance from surveillance spot to water in ArcGIS |  |  |  |
|  |  |  |  |  |  |  |  | water |  |  |  | negative: distance from surveillance spot to water |  |  |
|  |  |  |  |  |  |  | socioeconomics | community |  |  |  |  |  |  |
|  |  |  |  |  |  |  | geographic factors | altitude |  |  |  |  |  |  |
|  |  |  |  |  |  |  |  |  |  |  |  |  |  |  |
| CN | Li, W. H. [25] | 2014 | 7 districts of Guangzhou, Guangdong | DF cases | Guangdong CDC | district | LULC factors | agricultural land | vector data of base map and 2.5m SPOT5 | DF cases were spatially located | global Moran's I; Genetic Algorithm – Back Propagation (GA-BP) neural network model |  | negative |  |
|  |  |  |  |  |  |  |  | forest |  |  |  |  | negative |  |
|  |  |  |  |  |  |  |  | grassland |  |  |  |  | positive |  |
|  |  |  |  |  |  |  |  | orchard |  |  |  |  | positive |  |
|  |  |  |  |  |  |  |  | lake |  |  |  |  |  |  |
|  |  |  |  |  |  |  |  | ponds |  |  |  |  | negative |  |
|  |  |  |  |  |  |  |  | reservior |  |  |  |  | negative |  |
|  |  |  |  |  |  |  |  | rivers |  |  |  |  | negative |  |
|  |  |  |  |  |  |  |  | roads |  |  |  |  | positive |  |
|  |  |  |  |  |  |  |  | residential land |  |  |  |  | positive |  |
|  |  |  |  |  |  |  | air pollutants | SO2 | Agency of Environmental Protection of Guangzhou |  |  |  | positive |  |
|  |  |  |  |  |  |  |  | CO |  |  |  |  | negative |  |
|  |  |  |  |  |  |  |  | NO2 |  |  |  |  | negative |  |
|  |  |  |  |  |  |  |  | PM2.5 |  |  |  |  | positive |  |
|  |  |  |  |  |  |  |  | PM10 |  |  |  |  | negative |  |
|  |  |  |  |  |  |  | meteorological factors | max. temperature | Meteorological Bureau of Guangzhou |  |  |  | positive |  |
|  |  |  |  |  |  |  |  | min. temperature |  |  |  |  | positive |  |
|  |  |  |  |  |  |  |  | precipitation |  |  |  |  | negative |  |
|  |  |  |  |  |  |  |  | wind power |  |  |  |  |  |  |
|  |  |  |  |  |  |  |  | wind direction |  |  |  |  |  |  |
|  |  |  |  |  |  |  | socioeconomics | population density | not mentioned |  |  |  |  |  |
|  |  |  |  |  |  |  |  |  |  |  |  |  |  |  |
| CN | Yi, B. T. [26] | 1995 | Guangdong | BI | Guangdong CDC | city | LULC factors | NDVI | Remote sensing images from sensor AVHRR (advanced very high resolution radiometer) of satellite NOAA-14 (National Oceanic and Aeronautic Admission) | layouts of BI were made in ArcGIS | Tamhane's T2 multiple comparison test | positive |  |  |
|  |  |  |  |  |  |  |  |  |  |  |  |  |  |  |
| CN | Yue, Y. J. [27] | 2014 | 5 districts of Guangzhou, Guangdong | DF cases | NIDRIS | 1 km x 1 km | LULC factors | NDWI | Images and products of GF-1 | DF cases and environmental variables at gridded scale were calculated in ArcMap | Spearman correlation; multiple linear regression |  |  |  |
|  |  |  |  |  |  |  |  | land use type (LUT): water, vegetation, building |  |  |  | positive |  |  |
|  |  |  |  |  |  |  | meteorological factors | land surface temperature of daytime (LSTD) | MOD11A2 |  |  |  |  |  |
|  |  |  |  |  |  |  |  | land surface temperature of nighttime (LSTN) |  |  |  | positive |  |  |
|  |  |  |  |  |  |  |  | precipitable water vapor | MOD05 |  |  |  |  |  |
|  |  |  |  |  |  |  | socioeconomics | population | Institute of Geographic Sciences and Natural Resouces Research, CAS |  |  | positive |  |  |
|  |  |  |  |  |  |  |  | GDP |  |  |  |  |  |  |
|  |  |  |  |  |  |  |  |  |  |  |  |  |  |  |
| CN | Li, S. [28] | 2002 | Guangzhou, Guangdong | DF cases | Guangdong CDC | district | LULC factors | EVI | MOD13Q1 (vegetation index images of MODIS/TERRA) | EVI was calculated in ArcGIS | Pearson matrix correlation; OLS regression mode;, Enhanced principal component regression analysis |  | negative |  |
|  |  |  |  |  |  |  |  | small water body density (per km2) | SHPFILE of Guangzhou |  |  |  |  |  |
|  |  |  |  |  |  |  |  | urban construction land (covering rate) |  |  |  |  |  |  |
|  |  |  |  |  |  |  | meteorological factors | temperature | not mentioned |  |  |  |  |  |
|  |  |  |  |  |  |  |  | humidity |  |  |  |  |  |  |
|  |  |  |  |  |  |  |  | precipitation |  |  |  |  | positive |  |
|  |  |  |  |  |  |  |  | monthly sunlight |  |  |  |  |  |  |
|  |  |  |  |  |  |  |  | half-monthly cloud cover |  |  |  |  |  |  |
|  |  |  |  |  |  |  | socioeconomics | population density | Statistic yearbook |  |  |  | positive |  |

# Reference

1. Cao Z, Liu T, Li X, Wang J, Lin H, Chen L, et al. Individual and interactive effects of socio-ecological factors on dengue fever at fine spatial scale: A geographical detector-based analysis. International Journal of Environmental Research and Public Health. 2017;14(7):795. doi: 10.3390/ijerph14070795.

2. Li Q, Ren H, Zheng L, Cao W, Zhang A, Zhuang D, et al. Ecological niche modeling identifies fine-scale Areas at high risk of dengue fever in the Pearl River Delta, China. International Journal of Environmental Research and Public Health. 2017;14(6):619. doi: 10.3390/ijerph14060619.

3. Liu B, Gao X, Ma J, Jiao Z, Xiao J, Hayat MA, et al. Modeling the present and future distribution of arbovirus vectors Aedes aegypti and Aedes albopictus under climate change scenarios in Mainland China. Science of The Total Environment. 2019;664:203-14. doi: 10.1016/j.scitotenv.2019.01.301.

4. Liu K, Sun J, Liu X, Li R, Wang Y, Lu L, et al. Spatiotemporal patterns and determinants of dengue at county level in China from 2005–2017. International Journal of Infectious Diseases. 2018;77:96-104. doi: 10.1016/j.ijid.2018.09.003.

5. Qi X, Wang Y, Li Y, Meng Y, Chen Q, Ma J, et al. The effects of socioeconomic and environmental factors on the incidence of dengue fever in the Pearl River Delta, China, 2013. PLOS Neglected Tropical Diseases. 2015;9(10):e0004159. doi: 10.1371/journal.pntd.0004159.

6. Qu Y, Shi X, Wang Y, Li R, Lu L, Liu Q. Effects of socio-economic and environmental factors on the spatial heterogeneity of dengue fever investigated at a fine scale. Geospatial Health. 2018;13(2). doi: 10.4081/gh.2018.682.

7. Ren H, Wu W, Li T, Yang Z. Urban villages as transfer stations for dengue fever epidemic: A case study in the Guangzhou, China. PLOS Neglected Tropical Diseases. 2019;13(4):e0007350. doi: 10.1371/journal.pntd.0007350.

8. Ren H, Zheng L, Li Q, Yuan W, Lu L. Exploring determinants of spatial variations in the dengue fever epidemic using geographically weighted regression model: A case study in the joint Guangzhou-Foshan area, China, 2014. International Journal of Environmental Research and Public Health. 2017;14(12):1518. doi: 10.3390/ijerph14121518.

9. Tian H, Huang S, Zhou S, Bi P, Yang Z, Li X, et al. Surface water areas significantly impacted 2014 dengue outbreaks in Guangzhou, China. Environmental Research. 2016;150:299-305. doi: 10.1016/j.envres.2016.05.039.

10. Yue Y, Sun J, Liu X, Ren D, Liu Q, Xiao X, et al. Spatial analysis of dengue fever and exploration of its environmental and socio-economic risk factors using ordinary least squares: A case study in five districts of Guangzhou City, China, 2014. International Journal of Infectious Diseases. 2018;75:39-48. doi: 10.1016/j.ijid.2018.07.023.

11. Zheng L, Ren H-Y, Shi R-H, Lu L. Spatiotemporal characteristics and primary influencing factors of typical dengue fever epidemics in China. Infectious Diseases of Poverty. 2019;8(1):24. doi: 10.1186/s40249-019-0533-9.

12. Li Q, Cao W, Ren H, Ji Z, Jiang H. Spatiotemporal responses of dengue fever transmission to the road network in an urban area. Acta Tropica. 2018;183:8-13. doi: 10.1016/j.actatropica.2018.03.026.

13. Chuang T-W, Ng K-C, Nguyen T, Chaves L. Epidemiological characteristics and space-time analysis of the 2015 dengue outbreak in the metropolitan region of Tainan City, Taiwan. International Journal of Environmental Research and Public Health. 2018;15(3):396. doi: 10.3390/ijerph15030396.

14. Huang C-C, Tam T, Chern Y-R, Lung S-C, Chen N-T, Wu C-D. Spatial clustering of dengue fever incidence and its association with surrounding greenness. International Journal of Environmental Research and Public Health. 2018;15(9):1869. doi: 10.3390/ijerph15091869.

15. Chen T-HK, Chen VY-J, Wen T-H. Revisiting the role of rainfall variability and its interactive effects with the built environment in urban dengue outbreaks. Applied Geography. 2018;101:14-22. doi: 10.1016/j.apgeog.2018.10.005.

16. Hsueh Y-H, Lee J, Beltz L. Spatio-temporal patterns of dengue fever cases in Kaoshiung city, Taiwan, 2003–2008. Applied Geography. 2012;34:587-94. doi: 10.1016/j.apgeog.2012.03.003.

17. Wen T-H, Lin M-H, Fang C-T. Population movement and vector-borne disease transmission: Differentiating spatial–temporal diffusion patterns of commuting and noncommuting dengue cases. Annals of the Association of American Geographers. 2012;102(5):1026-37. doi: 10.1080/00045608.2012.671130.

18. Zheng L, Li Q, Ren H, Shi R, Bai K, Lu L. Exploring the relationship between dengue fever epidemics and social-environmental factors using land use regression model. Chinese Journal of Vector Biology and Control. 2018;29(3):226-34.

19. Li S, Tao H, Qin Y, Xu Y. Remote sensing and geoinformatics based environmental risk factors identification of dengue fever. Chinese Journal of Disease Control & Prevention. 2014;14(9):869-73.

20. Chen Y, Li W, Huang Y, Liang X. Rating system development of spatio-temporal diffusion risk factors on dengue fever based on random forests. Hubei Agricultural Sciences. 2017;56(7):1250-6.

21. Ren H, Wu W, Li Q, Lu L. Prediction of dengue fever based on back propagation neural network model in Guangzhou, China. Chinese Journal of Vector Biology and Control. 2018;29(3):221-5.

22. Yi B, Zhang Z, Xu D, Zhang B, Xi Y, Fu J, et al. Combined application of Co-Kriging and NDVI for studying the distribution of dengue fever in Guangdong province. Journal of Xi'an Jiaotong University (Medical Sciences). 2003;24(5):448-60.

23. Zheng L, Ren H, Shi R, Lu L. Spatial simulation and prediction of dengue fever transmission in Pearl River Delta. Journal of Geo-information Science. 2019;21(3):407-16.

24. Yi B, Xu D, Zhang Z, Zhang B, Xi Y, Fu J, et al. Development and application of geographic information system of Aedes vector in Chaozhou city, Guangdong Province. Chinese Journal of Epidemiology. 2004;25(2):134-7.

25. Li W, Chen Y, Wen L. Simulation of spatio-temporal diffusion of dengue fever based on the GA-BP neural network model. Journal of Image and Graphics. 2015;20(7):0981-91.

26. Yi B, Xu D, Zhang Z, Xi Y, Fu J, Luo J, et al. Study on the relationship between NDVI of NOAA-AVHRR image and the aedes density in Guangdong Province. Journal of Fourth Military Medical University. 2003;24(18):1720-4.

27. Yue Y, Lu L, Liu Q. Study on relationship between dengue fever epidemic and geographical environmental factors based on remote sensing geographic information system. Disease Surveillance. 2017;32(6):458-61.

28. Li S, Tao H, Xu Y. Study on spatial distribution modeling of dengue fever based on RS-GIS. Geomatics & Spatial Information Technology. 2008;31(5):56-9.
